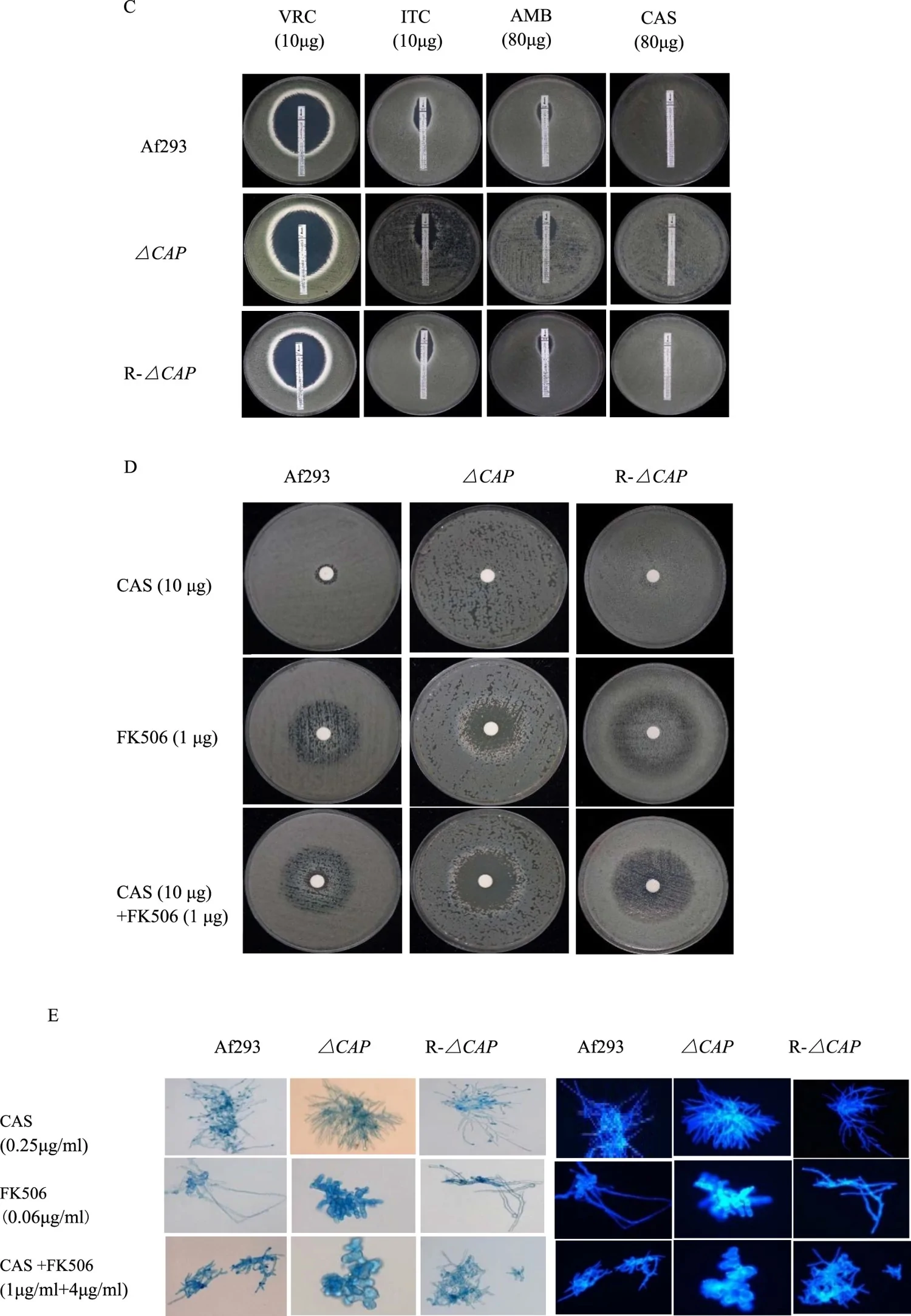# Correction

**DOI:** 10.1080/22221751.2026.2748212

**Published:** 2026-10-15

**Article title:** The cyclase-associated protein contributes to antifungal susceptibility and virulence in *Aspergillus fumigatus*

**Authors:** Yang X., Wang Q., Xiao B., Wang Q., Deng W., Osherov N., Li, R., Liu W.

**Journal:** *Emerging Microbes & Infections*

**Bibliometrics:** Volume 14, Issue 1

**DOI:** https://doi.org/10.1080/22221751.2025.2506795

When the article was initially published online, errors were identified in Figures 1 and 3. These errors have now been corrected in the online version. The correct figures are:

**Figure 1.**
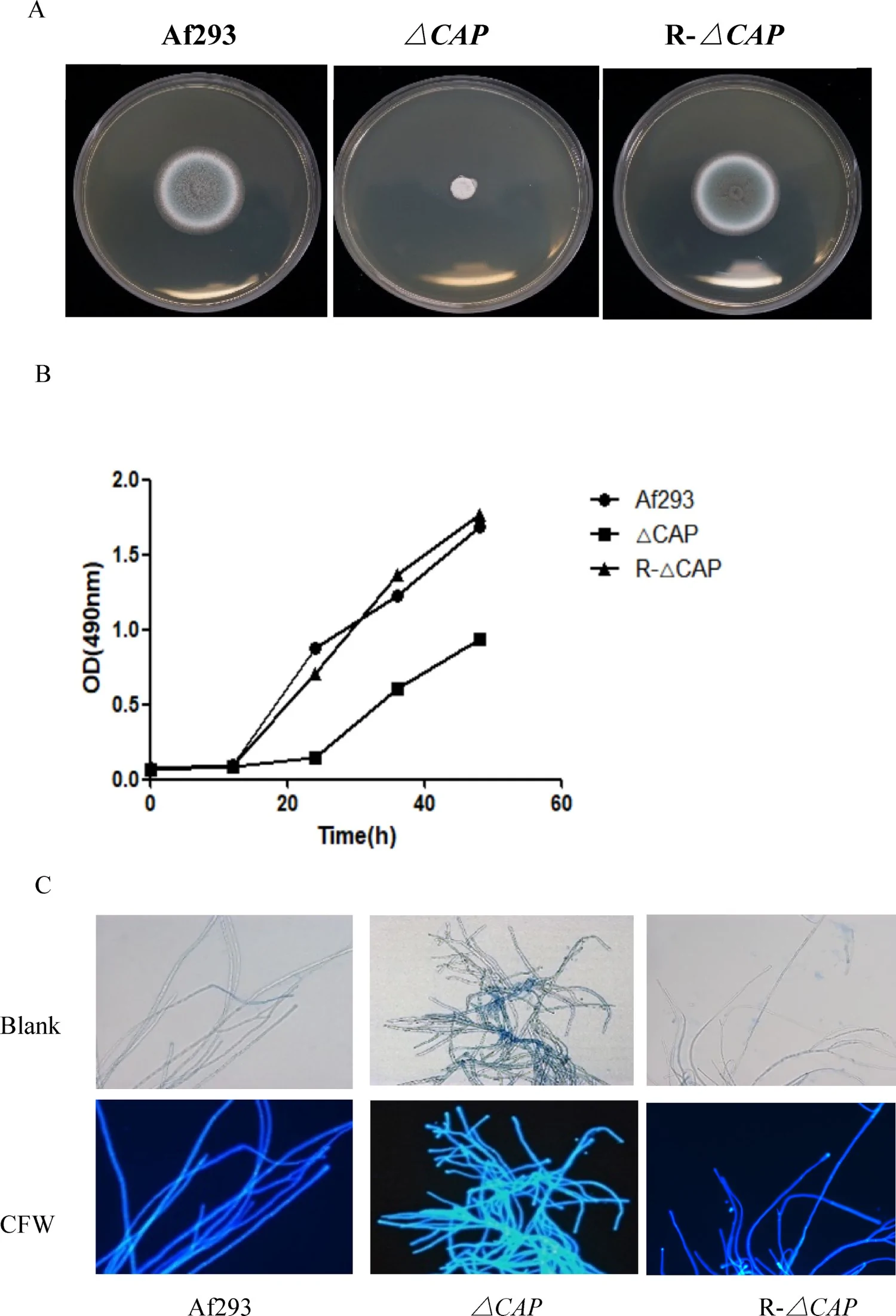
Growth rate and hyphal microscopic characteristics. (A) Colony size observation*.* (B) Growth rate detected by XTT assay. (C) Microscopic characteristics of hyphal morphology under white light and fluorescence microscopy following calcofluor white staining (×200 magnification).

Figure 3.Susceptibility to calcineurin inhibitors, triazoles, and the combination of FK506 and CAS. Susceptibility determined by (A) spot assay, (B) disk diffusion, (C) E-test assay. (D) Synergism with FK506 and CAS determined by disk diffusion assay. (E) Microscopic observations of hyphal morphology with CAS, FK506, and the combination of FK506 and CAS (200×magnification).

**Figure:**
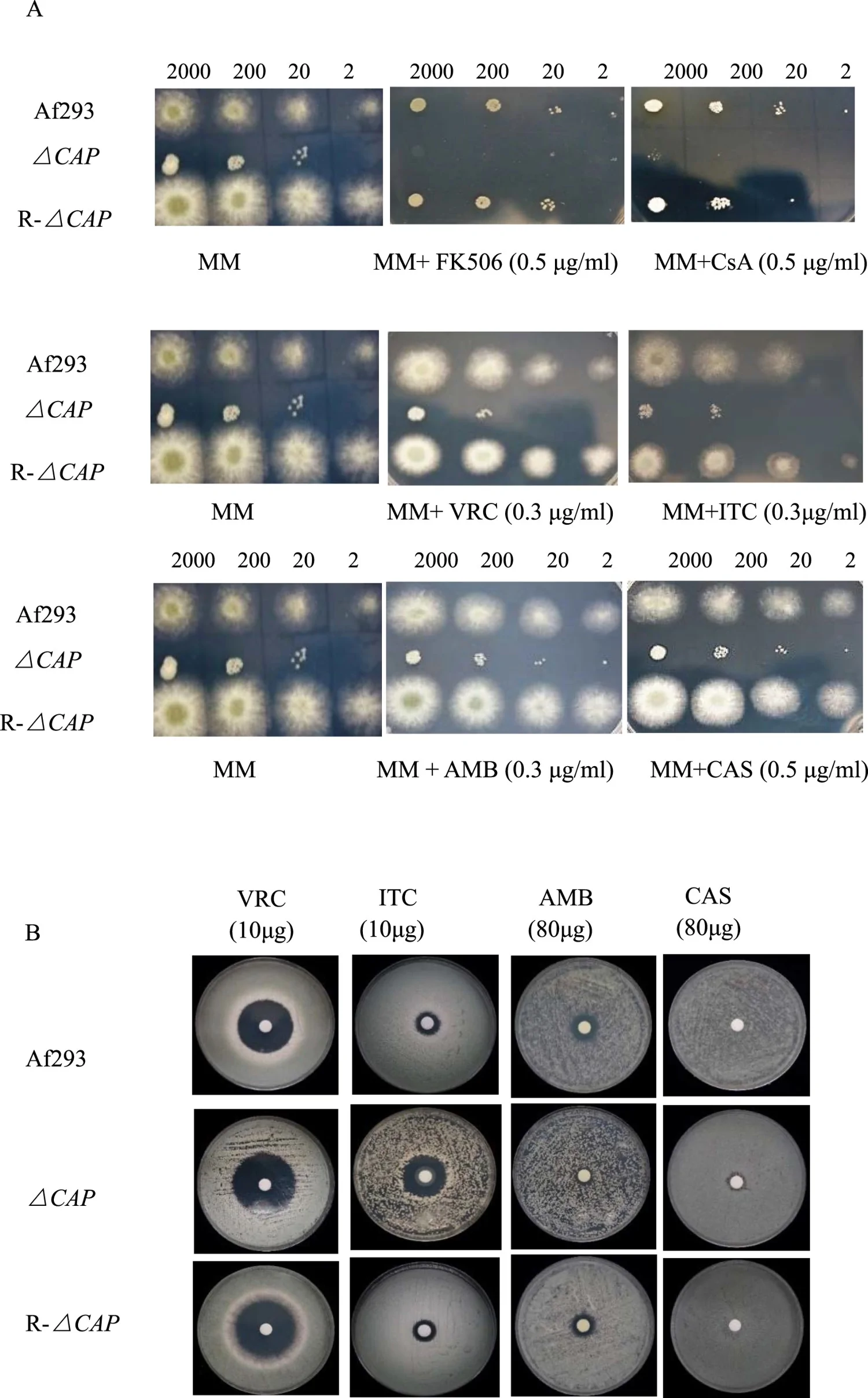

**Figure:**